# Do Immediate External Rewards Really Enhance Intrinsic Motivation?

**DOI:** 10.3389/fpsyg.2022.853879

**Published:** 2022-05-16

**Authors:** Yuxia Liu, Yang Yang, Xue Bai, Yujie Chen, Lei Mo

**Affiliations:** ^1^Key Laboratory of Brain, Cognition and Education Sciences, Ministry of Education, South China Normal University, Guangzhou, China; ^2^School of Psychology, South China Normal University, Guangzhou, China; ^3^School of Psychology, Center for Studies of Psychological Application, Guangzhou, China; ^4^School of Psychology, Guangdong Key Laboratory of Mental Health and Cognitive Science, Guangzhou, China

**Keywords:** immediate reward, external reward, intrinsic motivation, misattribution effect, temporal discounting

## Abstract

Researchers have conducted many studies on the relationship between external rewards and intrinsic motivation. A recent study showed that, compared with delayed rewards, rewards delivered immediately after the experiment enhanced the participants’ intrinsic motivation. However, this study did not rule out the possibility of a misattribution effect of extrinsic motivation. The present research conducted three studies to explore whether immediate rewards actually enhance intrinsic motivation. To rule out the interference of the misattribution effect of extrinsic motivation, according to the different characteristics of extrinsic motivation and intrinsic motivation, Study 1 and Study 2 improved the prior experimental paradigm, and the results indicated that the intrinsic motivation of participants who received extra rewards immediately after completing experimental tasks was stronger than that of participants who received the delayed extra reward. Furthermore, to rule out the potential interference of temporal discounting, Study 3 introduced a new variable—reward magnitude. The results showed that the delivery time of the extra reward had an independent effect on intrinsic motivation and that the immediacy of the extra reward could enhance intrinsic motivation. In all, the three studies strongly demonstrated that immediate external extra rewards could truly enhance intrinsic motivation.

## Introduction

In recent years, research on the influence of external rewards on intrinsic motivation has been conducted in social psychology, management psychology, and other fields. There are predominantly two perspectives on this issue. One perspective is the “declining account,” demonstrating that external rewards will undermine intrinsic motivation, as indicated by numerous empirical studies (Deci, 1971; Tang and Hall, 1995; Deci and Koestner, 1999; Maimaran and Fishbach, 2014). Another perspective is the “enhancing account,” which states that the undermining effect of external rewards on intrinsic motivation can be avoided and that external rewards may even enhance intrinsic motivation (Eisenberger and Cameron, 1996; Goswami and Urminsky, 2017). This perspective is also supported by several experimental studies, especially a recent study by Woolley and Fishbach (2018). The research found that giving extra rewards immediately after completing a task significantly promotes participants’ intrinsic motivation and raises their enthusiasm and interest in activities compared with delayed rewards, which firmly supported the “enhancing account” perspective.

Woolley and her colleagues attempted to clarify the influence of reward timing (immediate rewards vs. delayed rewards) on intrinsic motivation. The results showed that compared with delayed rewards or no rewards, paying extra rewards immediately may significantly enhance individuals’ intrinsic motivation (Woolley and Fishbach, 2018). Although their findings provided strong support for the “enhancing account” perspective that external incentives enhance intrinsic motivation, their study did not rule out the potential effect of the misattribution of extrinsic motivation. The misattribution effect of extrinsic motivation is that positive external stimuli (such as pleasant images, music, etc.) irrelevant to experimental tasks produce positive emotional experiences for people and actually increase extrinsic motivation. However, people sometimes mistakenly attribute this positive emotional experience to the enhancement of intrinsic motivation (Fishbach et al., 2004; Leander et al., 2017). Intrinsic motivation is triggered by the positive experience of the activity itself. However, in real life, people may not totally notice or remember subtle external rewards and may attribute the enhancement of external motivation directly to the activity itself. In other words, our perception of intrinsic motivation might instead be a byproduct of the misattribution effect of extrinsic motivation. Related studies have also confirmed that when an activity entails pleasant contextual clues, the participants’ perception of their intrinsic motivation will be significantly enhanced. Then, participants tend to report stronger intrinsic motivation than the baseline level in self-report measures (Leander et al., 2017). In Woolley’s study, participants who received a reward immediately after the experiment showed greater interest in the activity itself. We inferred that one possible explanation is that an immediate reward improved the intrinsic motivation level of the participants. Alternatively, it may also be that the participants’ extrinsic motivation improved due to the reinforcement of immediate rewards, but the enhancement of extrinsic motivation was mistakenly attributed to their interest in the activity itself, which was then reflected as higher scores of intrinsic motivation. Based on this analysis, it is necessary to rule out the possibility of a misattribution effect of extrinsic motivation and further explore how external rewards influence intrinsic motivation.

Prior studies on intrinsic and extrinsic motivation have suggested that the satisfaction of behaviors inspired by intrinsic motivation lies in the activity itself; thus, corresponding behaviors will be more persistent and stable and will not be easily influenced by external incentives. In contrast, extrinsic motivation may quickly and effectively change people’s behavior but may only be maintained for a short time. Additionally, behaviors driven by extrinsic motivation are generally passive. In this case, external incentives play a leading role in people’s actions, and task performance is easily affected by external incentives; thus, task performance may vary greatly.

We conducted three studies to systematically explore whether immediate rewards enhance the level of intrinsic motivation. We focused on the real influence of different external reward timing (immediate extra reward vs. delayed extra reward) on intrinsic motivation: Compared with the delayed extra reward, whether the immediate extra reward does enhance the intrinsic motivation, or it just result in a “false enhancement” of intrinsic motivation caused by the misattribution effect of extrinsic motivation.

According to the difference between the time-dependent characteristics of intrinsic motivation and extrinsic motivation, Study 1 included two subexperiments. In Study 1a, a replication experiment was conducted to demonstrate the reliability of the conclusion in Woolley’s study: immediate rewards enhance intrinsic motivation. To eliminate the possibility of the misattribution of extrinsic motivation, we improved the previous research paradigm and changed the measurement time of intrinsic motivation. However, Study 1b could not totally rule out the interference of the misattribution of extrinsic motivation. In Experiment 2, a new experimental scheme was implemented. Participants were asked to complete two reading tasks. Before the first reading task, the experimenters informed participants when they would receive an extra reward, but later, the participants were told that the delivery time of the extra reward changed temporarily. The intrinsic motivation of all participants was measured twice in Experiment 2.

Furthermore, according to some studies related to temporal discounting, earlier rewards actually increase extrinsic motivation because the same amount but earlier rewards may be psychologically larger due to temporal discounting (Ainslie and Haslam, 1992; Frederick et al., 2002). Other studies show that earlier rewards only increase intrinsic motivation because earlier rewards do not create an activity-goal fusion (Tang and Hall, 1995). Thus, temporal discounting is an important factor that may influence the experimental results of the present study. To explore the independent inference of reward timing on intrinsic motivation, in Study 3, we introduced the variable of reward magnitude and varied the magnitude and timing of extra rewards independently.

## Study 1

Study 1 aimed to rule out the possible misattribution effect of extrinsic motivation and explore whether immediate extra rewards can enhance participants’ intrinsic motivation. Study 1 included two subexperiments: Study 1a and Study 1b. Study 1a used the experimental paradigm of Woolley’s study to demonstrate the reliability of prior conclusions: immediate rewards can enhance intrinsic motivation. Considering the potential interference of misattribution of extrinsic motivation, Study 1b changed the measuring time of intrinsic motivation from before the delivery of immediate extra rewards (in Study 1a) to before the delivery of delayed extra rewards, to further explore how the extra reward delivery time influences intrinsic motivation.

### Study 1a

A recent study by Woolley et al. showed that the delivery time of rewards influenced intrinsic motivation and that immediate rewards could increase intrinsic motivation. Drawing on the research paradigm of Woolley et al., Study 1a aimed to demonstrate the reliability of the conclusion that immediate rewards could enhance intrinsic motivation.

#### Method

##### Participants

A priori power analysis carried out using G*Power software (Faul et al., 2007), indicated that to detect a large effect-size of *d* = 0.4, for the single-factor ANOVA, with an alpha of 0.05 and power = 0.80, a sample of 66 participants would be needed. Ninety participants took part in this experiment, and three of them had read the experimental extracts before, so they were ruled out. Among the remaining 87 participants, 59% were women aged between 17 and 26 years old (M = 19.2, SD = 1.41). All participants came from South China Normal University with normal or correct-to-normal vision. The experimental protocols were approved by the Human Research Ethics Committee for Non-Clinical Faculties of The School of Psychology, South China Normal University. Informed consent was obtained from all subjects.

##### Materials

###### Adapted Intrinsic Motivation Inventory

Combined with the present study, we adapted the Intrinsic Motivation Inventory (IMI) revised by Woolley and Fishbach (2018) and used the new inventory to measure the intrinsic motivation level of participants. This inventory measured the intensity of participants’ intrinsic motivation in reading activities from the interest-enjoyment dimension, including six items. For example, one item description of this inventory is, “How much did you enjoy the content in this article?” Participants are asked to rate the content on a seven-point scale (1 means “not at all” and 7 means “very much”). This scale also set 1 item to measure the behavioral aspects of intrinsic motivation to determine the extent to which participants would choose to continue engaging in the focal task. In summary, there were seven items in the adapted IMI used in the present study. The confirmatory factor analysis of the scale showed that the fitting indices were *x*^2^/*df* = 3.08, RMSEA = 0.08, and CFI = 0.96. The Cronbach’s α coefficient of the scale was 0.83, meeting the measurement standard (see Appendix A in Supplementary Material for details). The average score of all items was calculated as the intrinsic motivation score.

###### Reading Materials

The reading materials in the reading task were extracted from Liang Shiqiu’s translation of Meditations.

#### Procedure

This experiment was a single-factor between-subjects design. The independent variable was extra reward timing (immediate extra reward vs. delayed extra reward vs. no extra reward). The dependent variable was intrinsic motivation.

**FIGURE 1:**
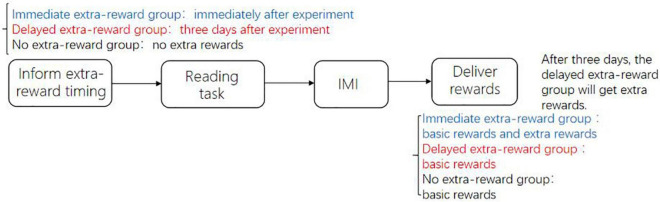
Experimental procedure of Study 1a.

First, participants were randomly assigned to three groups: two were the experimental group (the immediate extra reward group, the delayed extra reward group), and one was the control group (the no extra reward group), with 29 people in each group. Before the experiment started, the process of the experiment was explained to all participants (see Figure 1): they would do a reading task of specific reading materials. Participants were required to read the materials carefully and answer the questions related to the materials after reading. Then, they completed an adapted IMI to measure their intrinsic motivation. Additionally, participants in the two experimental groups were told that except for basic rewards, they would receive an extra reward, but the delivery time of the extra reward was different. Participants in the immediate extra reward group were told that they would receive an extra reward of 5 RMB immediately after they finished the experiment, while participants in the delayed extra reward group were told that they would receive an extra reward of 5 RMB 3 days after they finished the experiment. Participants in the control group had no extra reward. All participants in the three groups received the same amount of basic reward at the same time (after they completed the adapted IMI).

In the present study, the independent variable of the different experimental groups is the delivery time of extra reward. We gave all participants a basic reward for their participation in the experiments. The basic reward was delivered at the same time (after completing the first IMI) under different conditions.

#### Results

**FIGURE 2:**
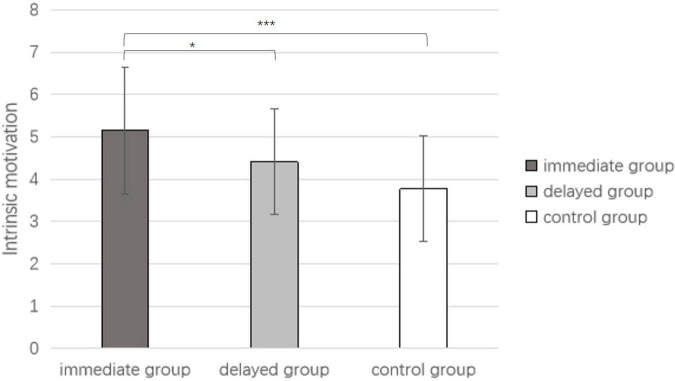
IMI scores under the conditions of immediate extra reward, delayed extra reward, and no extra reward (In the bar graph above, the error bars represent the standard deviations; * represents *p* < 0.05, ^***^ represents *p* < 0.001).

The results for the immediate extra reward group are M = 5.15, SD = 1.50; for the delayed extra reward group they are M = 4.41, SD = 1.25; and for the no extra reward group they are M = 3.77, SD = 1.25. The participants’ intrinsic motivation scores of the three groups were statistically tested by single-factor ANOVA. The results showed that the main effect of the extra reward timing was significant [*F*_(2,84)_ = 7.659, *p* = 0.001, $\eta_{p}^{2}$ = 0.15, see Figure 2]. The results of multiple comparisons indicated that the intrinsic motivation scores of the immediate extra reward group were significantly different from those of the delayed extra reward group (*p* = 0.039) and the control group (*p* < 0.01). There was no significant difference in intrinsic motivation scores between the delayed-reward group and the control group (*p* = 0.073).

#### Discussion

The results of Study 1a were consistent with the previous study of Woolley, which preliminarily indicated that, compared with the delayed extra rewards and no extra rewards, immediate rewards could increase intrinsic motivation. However, the measured scores of the intrinsic motivation of participants in the immediate extra reward group were higher than those in the other groups. It could not be confirmed that the immediate extra reward enhanced intrinsic motivation because participants might mistakenly attribute the enhancement of extrinsic motivation to that of intrinsic motivation. Therefore, it was necessary to implement another experiment to eliminate the interference of the misattribution effect of extrinsic motivation.

### Study 1b

Study 1a did not rule out the interference of the misattribution of extrinsic motivation. According to the time-dependent characteristics of intrinsic motivation and extrinsic motivation (intrinsic motivation is stable, while extrinsic motivation can decrease quickly over time), Study 1b improved the previous research paradigm (changing the measuring time of intrinsic motivation) to address whether immediate extra rewards would truly increase intrinsic motivation.

#### Method

##### Participants

A priori power analysis carried out using G*Power software (Faul et al., 2007) indicated that to detect a large effect size of *d* = 0.8, for the independent sample *t*-test, with an alpha of 0.05 and power = 0.80, a sample of 42 participants would be needed. Fifty-eight participants took part in this experiment, and 63% were women aged between 17 and 26 years old (M = 20.1, SD = 1.72). All participants came from South China Normal University with normal or correct-to-normal vision. The experimental protocols were approved by the Human Research Ethics Committee for Non-Clinical Faculties of School of Psychology, South China Normal University. Informed consent was obtained from all subjects.

##### Materials

###### Adapted Intrinsic Motivation Inventory

Same as Study 1a.

###### Reading Materials

The reading material in the first reading task was the same as in Study 1a. The reading material of the second reading task was different from that of the first. However, it was taken from the same book as the first reading task, and the level of reading difficulty as well as reading pleasure were balanced between the two tasks.

#### Procedure

This experiment was a single-factor between-subjects design. The independent variable was reward timing (immediate extra reward vs. delayed extra reward), and the dependent variable was intrinsic motivation. The index of the dependent variable was the score of the adapted Intrinsic Motivation Inventory.

**FIGURE 3:**
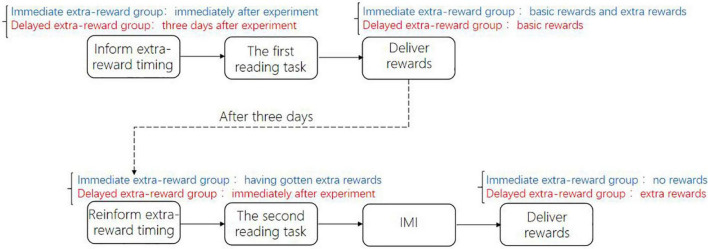
Experimental procedure of Study 1b.

First, participants were randomly assigned to the immediate or delayed extra reward, with 29 people in each group. Before the experiment started, all participants were informed of the process of the experiment (see Figure 3): they would first carry out the reading task of specific reading materials and then complete the first adapted IMI. The basic rewards would be paid after participants completed the first IMI. Then, the participants in two groups were informed that they needed to return to the laboratory 3 days later to complete the second reading task. Additionally, before the experiment started, participants in the immediate extra reward group were told that they would receive an extra reward of 5 RMB immediately after completing the first IMI, while participants in the delayed extra reward group were told that they would receive an extra reward of 5 RMB 3 days after completing the second reading task. Participants in the two groups received the same amount for the basic reward and extra rewards.

Three days later, when the participants in the immediate extra reward group had already received the extra rewards and participants in the delayed extra reward group were about to receive their extra rewards, all participants were asked to complete a second reading task. Before the second reading task started, participants in the immediate extra reward group were reminded that they would not receive any reward after finishing the whole experiment. Participants in the delayed extra reward group were told that they would receive extra rewards immediately after finishing the whole experiment. After completing the second reading task, all participants’ intrinsic motivation levels were measured simultaneously by the adapted IMI. After completing the adapted IMI, participants in the delayed extra reward group immediately received the extra reward.

#### Results

**FIGURE 4:**
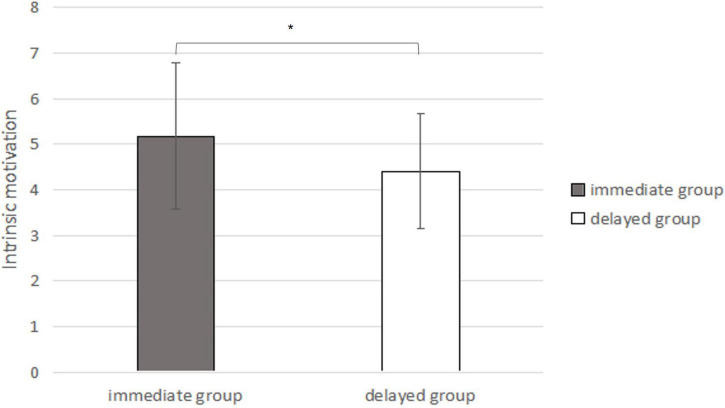
IMI scores under the conditions of immediate extra rewards and delayed extra rewards (In the bar graph above, the error bars represent the standard deviations; * represents *p* < 0.05).

The intrinsic motivation scores of the immediate extra reward group were M = 5.18, SD = 1.61, and those of the delayed extra reward group were M = 4.41, SD = 1.27. Independent sample *t*-tests showed that the intrinsic motivation scores of the immediate reward group and delayed reward group were significantly different [*t*_(56)_ = 2.018, *p* = 0.048 (α = 0.05), 95% CI_*diff*_ = [0.01,1.53], *d* = 0.54, see Figure 4]. Consistent with the results of Study 1a, the intrinsic motivation scores of the immediate extra reward group were still higher than those of the delayed extra reward group.

#### Discussion

According to the short-term nature of extrinsic motivation (He Kekang et al., 2002; Maimaran and Fishbach, 2014), if only the misattribution effect of extrinsic motivation exists, that is, participants misattributed the enhancement of extrinsic motivation to that of intrinsic motivation, in Study 1b, the intrinsic motivation scores of the participants in the delayed extra reward group would be higher than those in the immediate extra reward group. At the time point of measuring intrinsic motivation in Study 1b, the delayed extra reward group would receive the extra rewards immediately, while the immediate extra reward group did not have any rewards. However, the results of Experiment 1b were not consistent with this hypothesis. After changing the measurement timing of intrinsic motivation, the intrinsic motivation scores in the immediate extra reward group were still significantly higher than those in the delayed-reward group, which indicated that immediate extra rewards might increase intrinsic motivation. Study 1b further indicated that the immediate bonus could genuinely enhance the intrinsic motivation of the participants. However, Study 1b could not totally rule out the possibility of a misattribution effect of extrinsic motivation.

## Study 2

Study 2 created a new experimental scheme to further clarify how the immediate extra rewards influence intrinsic motivation.

### Method

#### Participants

A priori power analysis carried out using G*Power software (Faul et al., 2007) indicated that to detect a large effect size of *d* = 0.8, for the independent sample *t*-test, with an alpha of 0.05 and power = 0.80, a sample of 42 participants would be needed. Sixty-three participants took part in this experiment, and five of them had read the experimental extracts before, so they were ruled out. Among the remaining 58 participants, 56% were women aged between 17 and 26 years old (M = 19.7, SD = 1.73). All participants came from South China Normal University with normal or correct-to-normal vision. The experimental protocols were approved by the Human Research Ethics Committee for Non-Clinical Faculties of The School of Psychology, South China Normal University. Informed consent was obtained from all subjects.

#### Materials

Same as Study 1b.

### Procedure

This experiment was a single-factor between-subjects experimental design. The independent variable was the extra reward timing (immediate extra rewards vs. delayed extra rewards), and the dependent variable was intrinsic motivation.

**FIGURE 5:**
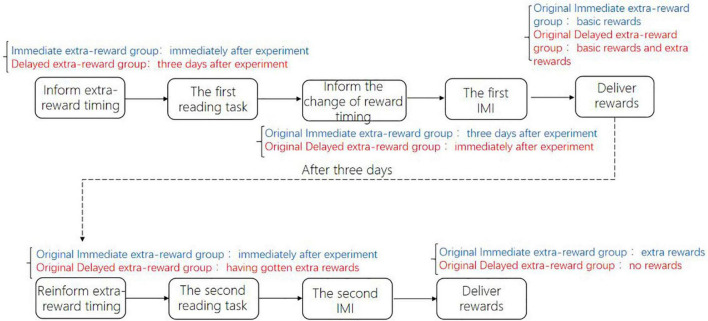
Experimental procedure of Study 2.

First, the participants were randomly assigned to the immediate extra reward group or the delayed extra reward group, with 29 people in each group. Before the experiment started, the experiment was explained to all participants (see Figure 5): they would carry out a reading task of specific reading materials and then complete the adapted IMI. The basic rewards would be paid after participants completed the first IMI. Additionally, before the experiment started, participants in the immediate extra reward group were told that they would receive an extra reward of 5 RMB immediately after completing the IMI, while participants in the delayed extra reward group were told that they would receive an extra reward of 5 RMB 3 days later.

After completing the reading task, participants in the immediate extra reward group were told that for some reason, the extra reward could not be paid until 3 days later. Conversely, participants in the delayed extra reward group were told that they would receive the extra reward immediately after completing the IMI. Then, the adapted Intrinsic Motivation Inventory was used to measure the intrinsic motivation of all participants simultaneously.

Due to the sudden change in the timing of the extra rewards, participants’ emotions might be badly affected, which might further decrease their performance in completing follow-up tasks. To eliminate this possibility, we used a questionnaire to evaluate the influence of changing reward timing on follow-up task performance as well as the main standard of the participants’ rating (see Appendix B in Supplementary Material for details).

We used the term “original immediate extra reward group” to represent the group that was first informed of receiving the extra reward immediately after completing the IMI but was later told they would not receive the extra reward until 3 days later. Likewise, the term “original delayed extra reward group” designated the group that was first informed of receiving the extra reward 3 days after completing IMI but was later told they would receive the extra reward immediately upon completing the IMI.

After the questionnaire was completed, participants in the original delayed extra reward group received the basic rewards as well as the extra rewards, while participants in the original immediate extra reward group only received the basic rewards. At the same time, the experimenters told the participants of the two groups to return to the laboratory 3 days later to perform the second reading task.

Three days after the participants finished the first reading task, that is, when the participants in the original delayed extra reward group had already received the extra reward, and the participants in the original immediate extra reward group were about to receive the extra reward, participants in the two groups were asked to complete the second reading task. Then, the intrinsic motivation intensity of the two groups was measured for the second time. Before the task started, the experimenters told participants in the original immediate-reward group that they would receive extra rewards immediately after completing the tasks, while participants in the original delayed-reward group were told that they would not receive any reward after completing the tasks. After completing the reading task, the Intrinsic Motivation Inventory was used to measure the intrinsic motivation of all participants the second time. After completing the IMI, participants in the original immediate-reward group received the extra reward.

### Results

#### Questionnaire Results

The questionnaire data of the original immediate-reward group showed that one participant thought that changing the reward timing would influence his performance in subsequent tasks. This participant’s second measurement result did not change compared with the first task; thus, the experimental data of this participant were not excluded. The remaining 28 participants thought that changing the reward timing had no effect on their subsequent task, and their scoring basis mainly included the following three points. (1) A total of 82.1% of the participants’ scoring was based on the subjective recognition of the reading content. They thought that the reading content was intriguing and enlightening, and the reading activity itself was satisfying, so they mainly scored according to their personal preference. (2) A total of 17.9% of the participants believed that they should obey the experimental rules and complete the task carefully, which is irrelevant to the magnitude and timing of the reward. (3) A total of 14.3% of the participants thought that the reward would be available sooner or later, and they did not have an urgent need for money; thus, changing the reward timing had little effect on them.

The questionnaire survey data of the original delayed-reward group showed that four participants thought that changing the reward timing would have an impact on their subsequent scoring: among them, the second measurement of two participants did not change compared with the first; the second measurement score of one subject decreased by 0.25 compared with the first one; and the second measurement score of another subject was 0.75 lower than that of the first (M = 4.78, SD = 1.61). The score differences were all within the range of one standard deviation; thus, the experimental data of these four participants were retained, and the first measurement results were used for the final calculation. The remaining 24 participants thought that changing the reward timing would not influence their subsequent scores. (1) Among them, 45.8% of the subjects scored according to their subjective feelings when reading. They thought that the reading content was attractive and instructive, and the reading activity itself was enjoyable. (2) A total of 37.5% of the participants thought they should obey the experimental rules and complete the task carefully, which is irrelevant to the magnitude and timing of the reward. (3) A total of 29.2% of the participants thought that the reward would be available sooner or later, and they did not have an urgent need for money; thus, changing the reward timing had little effect on them.

Comparing the results of the questionnaire between the two groups, we concluded that compared with the original delayed-reward group (45.8%), participants in the original immediate-reward group (82.1%) had greater recognition of the reading content and found the reading activities more enjoyable.

#### Scores of the First Intrinsic Motivation Inventory

**FIGURE 6:**
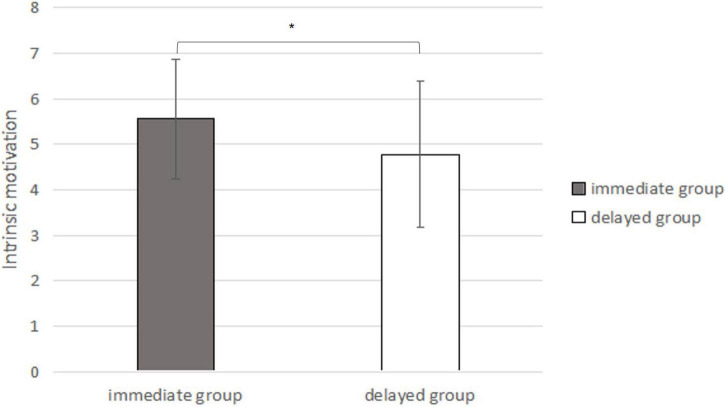
First IMI scores under the original immediate extra reward and original delayed extra reward conditions (In the bar graph above, the error bars represent the standard deviations; * represents *p* < 0.05).

The first intrinsic motivation scores of the original immediate extra reward group were M = 5.56, SD = 1.32, and the first intrinsic motivation scores of the original delayed extra reward group were M = 4.78, SD = 1.61. An independent sample *t*-test showed that the first intrinsic motivation scores of the original immediate-reward group and the original delayed-reward group were significantly different [*t*_(56)_ = 2.022, *p* = 0.048, 95% CI_*diff*_ = [0.01, 1.56]; *d* = 0.54, see Figure 6].

#### Scores of the Second Intrinsic Motivation Inventory

**FIGURE 7:**
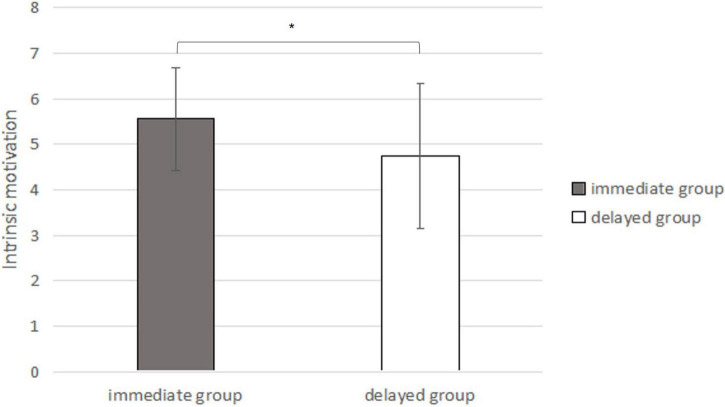
The second IMI scores under the conditions of original immediate extra reward and original delayed extra reward (In the bar graph above, the error bars represent the standard deviations; * represents *p* < 0.05).

The second intrinsic motivation scores of the original immediate-reward group were M = 5.56, SD = 1.13, and the second intrinsic motivation scores of the original delayed reward group were M = 4.74, SD = 1.60. Independent sample *t*-tests showed that the second intrinsic motivation scores of the original immediate-reward group and the original delayed-reward group were significantly different [*t*_(56)_ = 2.274, *p* = 0.027, 95% CI_*diff*_ = [0.10, 1.56]; *d* = 0.61, see Figure 7].

The first intrinsic motivation scores of participants in the original immediate extra reward group were compared with the scores of second IMI. The first intrinsic motivation scores of the original immediate extra reward group were M = 5.56 and SD = 1.32, while the scores of second IMI were M = 5.56 and SD = 1.13. The paired sample *t*-test showed that the difference between the two intrinsic motivation scores was not statistically significant [*t*_(28)_ = 0.014, *p* = 0.98].

### Discussion

The results of Study 2 illustrated that although the delivery time of the extra reward was suddenly changed after participants completed the first reading task, the results of the first IMI scores showed that the intrinsic motivation of the original immediate extra reward group was significantly stronger than that of the original delayed extra reward group. The results of the second IMI scores also showed that the intrinsic motivation of the original immediate extra reward group was stronger than that of the original delayed extra reward group. Furthermore, scores of the first and the second IMI of participants in the original immediate extra reward group did not have a significant difference, which indicated that there was no misattribution effect of extrinsic motivation. If the misattribution effect of extrinsic motivation exists, at the time point of the first IMI, when the original immediate extra reward group would not receive any reward, the IMI scores would be lower than the second IMI when the participants would receive the extra reward. Overall, study 2 efficiently ruled out the interference of the misattribution effect of extrinsic motivation and provided new evidence for the conclusion that “immediate extra reward can enhance intrinsic motivation.”

## Study 3

Considering the potential interference of temporal discounting, Study 3 introduced a new variable, reward magnitude, to further explore the independent influence of the time of the delivery of extra rewards on intrinsic motivation. We assumed that, compared with the larger rewards, immediate reward delivery had a stronger effect on intrinsic motivation.

### Method

#### Pilot Test

To compare the influence of reward timing and reward magnitude on intrinsic motivation, it was necessary to modify the amount of the delayed bonus to make it the same level as or slightly higher than the amount of the immediate bonus in participants’ subjective feelings. In the pilot test, a questionnaire survey was used to calculate the temporal discount of reward magnitude. Ninety-seven students (M_*age*_ = 23.4 years old, SD = 1.30) in South China Normal University participated in the experiment; among them, 57% were women.

The matching task paradigm proposed by Richard (1981) was adopted in the pilot test. In this paradigm, the experimenters first fill in the reward amount (the amount is small) that can be paid immediately and then ask the subjects to fill in the amount they have in mind that is equivalent to the immediate reward if the reward will be paid after a period of delay (Jiang et al., 2014). The purpose of this paradigm is to make the perceived value of rewards delivered at two different times close to or equal to each other. Drawing from this paradigm, the present study established the reward amount that could be delivered immediately as 5 RMB and asked the participants to answer the question: “If you had to wait 3 days to get the reward, how much money would you accept?” Previous studies on risk aversion showed that change of the degree of regret over time typically presented as a logarithmic distribution (Gandelman and Hernández-Murillo, 2013; Vakili and Zhao, 2016). Therefore, here we used a geometric mean to estimate the amount of delayed reward equivalent to the immediate reward.

The survey results indicated that the geometric average was 5.46 RMB. Thus, in Study 3, the amount of the low-reward group was set as 5 RMB, and the amount of the high-reward group was set as 7 RMB to ensure that the attraction of the delayed reward to the participants was no less than that of the immediate reward. On this basis, we aimed to clarify whether immediate but small rewards could increase participants’ intrinsic motivation more than delayed but large rewards.

#### Participants

A priori power analysis carried out using G*Power software (Faul et al., 2007) indicated that to detect a large effect-size of *d* = 0.4, for the two-factor ANOVA, with an alpha of 0.05 and power = 0.80, a sample of 73 participants would be needed. A total of 144 participants took part in this experiment, of whom 56% were women aged between 17 and 26 years old (M = 21.2, SD = 1.73). All participants came from South China Normal University with normal or correct-to-normal vision. The experimental protocols were approved by the Human Research Ethics Committee for Non-Clinical Faculties of School of Psychology, South China Normal University. Informed consent was obtained from all subjects.

#### Materials

Same as Study 1a.

### Procedure

A two-factor between-subjects experimental design was conducted in this experiment. The independent variables were the timing (immediate extra rewards vs. delayed extra rewards) and magnitude (high vs. low) of the reward. The dependent variable was the intrinsic motivation level.

**FIGURE 8:**
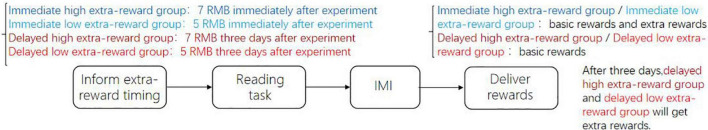
Experimental procedure of Study 3.

First, participants were randomly assigned to four groups: the immediate and high extra reward group, the immediate and low extra reward group, the delayed and high extra reward group, and the delayed and low extra reward group, with 36 people in each group. Before the experiment started, the experiment was explained to all participants (see Figure 8): they would do a reading task of specific reading materials. Participants were required to read the materials carefully and answer the questions related to the materials after reading. Then, they completed an adapted IMI to measure their intrinsic motivation. Additionally, before the experiment started, participants in the immediate and high extra reward group as well as the immediate and low extra reward group were told that they would receive an extra reward immediately after they completed the IMI, while participants in the delayed and high extra reward group as well as the delayed and low extra reward group were told that they would receive an extra reward 3 days after they completed the IMI.

### Results

**FIGURE 9:**
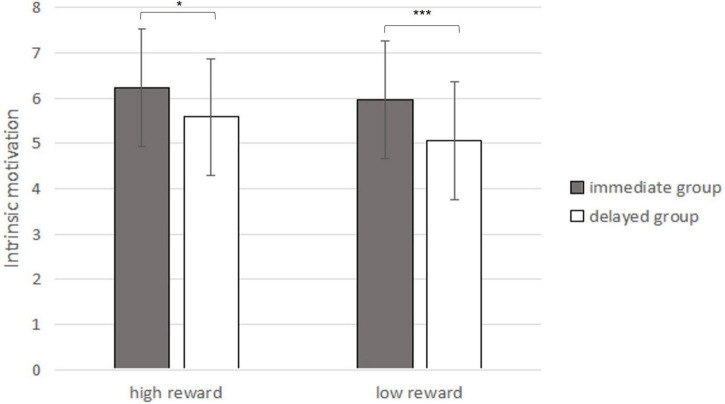
Influence of extra reward timing and extra reward magnitude on intrinsic motivation (In the bar graph above, the error bars represent the standard deviations; * represents *p* < 0.05, ^***^ represents *p* < 0.001).

The data of the experiment were statistically tested by ANOVA with a two-factor completely random design. The results indicated that the interaction between the two independent variables (extra reward timing and extra reward magnitude) was not significant [*F*_(1,140)_ = 0.353, *p* = 0.553, see Figure 9]. The main effect of extra reward timing was significant [*F*_(1,140)_ = 13.670, *p* = 0.001, $\eta_{p}^{2}$ 0.09]. The intrinsic motivation score of the immediate-reward group was significantly higher than that of the delayed-reward group (M_*immediate*_ = 6.10, SD = 0.99; 5.33, M_*delay*_ = 5.33, SD = 1.50). The main effect of reward magnitude was not significant [*F*_(1,140)_ = 3.417, *p* = 0.067]. There was no significant difference between the intrinsic motivation scores of participants in the large-reward group and those in the small-reward group (M_*large*_= 5.91, SD = 1.15;}M_*small*_ = 5.52, SD = 1.46).

### Discussion

The results of Study 3 illustrated that, compared with a low extra reward magnitude, a larger extra reward could not significantly enhance participants’ intrinsic motivation. For the factor of extra reward timing, the immediate extra reward could significantly increase participants’ intrinsic motivation compared with the delayed reward, which indicated that immediate rewards increased intrinsic motivation and that it was not caused by the subjective higher value of immediate rewards but rather the immediacy. Study 3 eliminated the interference of the temporal discounting effect and further verified the enhancement effect of immediate extra reward on intrinsic motivation.

## General Discussion

In the present study, three studies were carried out to eliminate the interference of the misattribution effect of extrinsic motivation as well as temporal discounting in an attempt to clarify the influence of external extra reward timing on intrinsic motivation.

In Study 1, the results in Study 1a were consistent with previous research. However, the experimental method of Study 1a was not able to rule out the interference of the misattribution effect of extrinsic motivation. To answer this question, Study 1b changed the time point of measuring intrinsic motivation. The results of Study 1b were consistent with those of Study 1a. Although the delayed extra reward group received extra rewards immediately after the second reading task, the intrinsic motivation scores of the participants in the delayed extra reward group were still lower than those in the immediate-reward group.

For Study 2, the results illustrated that although the delivery time of the extra reward was suddenly changed after participants completed the first reading task, the results of the two IMI scores both indicated that the intrinsic motivation of the original immediate extra reward group was significantly stronger than that of the original delayed extra reward group. Furthermore, there was no significant difference between the two IMI scores of participants in the original immediate extra reward group, which indicated that there was no misattribution effect of extrinsic motivation. Overall, Study 2 efficiently ruled out the interference of the misattribution effect of extrinsic motivation and provided new evidence that immediate extra rewards can enhance intrinsic motivation.

Study 3 excluded the potential interference of temporal discounting. The results showed no interaction between the timing or magnitude of extra rewards, and the main effect of extra reward magnitude was not significant, while extra reward timing had a significant impact on intrinsic motivation intensity. This result confirmed the independent influence of immediate rewards on intrinsic motivation.

In conclusion, based on Woolley and her colleagues’ research, the present study efficiently ruled out the potential interference of the misattribution effect of extrinsic motivation as well as temporal discounting and demonstrated that delivering an immediate extra reward could enhance the intrinsic motivation level.

The results of the present study were consistent with research concerning evaluative conditioning (EC). Researchers usually associate a conditioned stimulus (CS) with a positive or negative unconditioned stimulus (US) so that participants will form a positive or negative attitude toward the original neutral stimulus. That is, people’s preference for a neutral stimulus can be changed by pairing it with another stimulus that people like or dislike. When an activity relates to a specific reward, even if the reward is canceled later, the previously formed connection between the two can still promote the individual’s enthusiasm for the activity (Razran, 1954; De Houwer et al., 2001). For example, when pairing a strange person with a pleasant picture, people are more likely to make positive comments about the stranger, while when another stranger is paired with negative pictures, people are more likely to make negative comments about that stranger (Rozin and Zellner, 1985; De Houwer, 2007; Hofmann et al., 2010). Moreover, in the current study, the delivery of extra rewards was not based on specific performance criteria, such as perfect task performance. From the studies on positive affect effects, this kind of non-contingent reward primarily induces positive affect, while a specifically performance-contingent reward has a motivational effect. For example, Müller et al. (2007) found that rewards perceived by participants as an easy gain had the same effect as showing positive affective pictures (without any reward). Furthermore, Fröber and Dreisbach (2014, 2016) demonstrated that only performance-contingent, but not non-contingent, rewards have the motivational effect of increasing proactive control, whereas non-contingent rewards decrease proactive control, similar to a non-reward positive effect manipulation. Additionally, in older studies on positive affect effects, giving participants a surprise reward as a gift was a common method to induce positive affect (Ashby et al., 1999). Therefore, the effects demonstrated in the present study might also be mediated by the positive affect induced by the extra reward, which was unconditional. In addition, relevant studies have shown that the shorter the time interval between the conditional stimulus and unconditional stimulus, the stronger the conditioning effect (Balsam et al., 2010; Boakes and Costa, 2014). The present study illustrated that, compared with the delayed reward, an immediate reward could significantly increase participants’ interest and enthusiasm for tasks and effectively stimulate participants’ intrinsic motivation for tasks. This measuring result of intrinsic motivation was consistent with the theoretical explanation of evaluative conditioning.

Additionally, the findings of the present study were related to the studies on the impact of immediate rewards on goal persistence, which showed that, compared with the delayed-reward condition, immediate rewards had a stronger correlation with the permanence of individuals’ participation in activities (Volpp et al., 2008; John et al., 2011; Acland and Levy, 2015; Woolley and Fishbach, 2016). The research on long-term goals found that paying attention to immediate returns in the process was more helpful to improve the durability of these long-term goals than paying attention to long-term and delayed returns (Woolley and Fishbach, 2016). For example, compared with the long-term goal of improving health through exercise, combining immediate rewards (such as listening to an engaging novel) with physical exercise could significantly increase the individuals’ exercise frequency (Milkman et al., 2014). The measuring results of intrinsic motivation in the present study were consistent with the research concerning goal persistence. Furthermore, research on how to offset self-control consumption found that engaging in a consumptive task, linking the task with an economic reward (Boksem et al., 2006), or giving participants an immediate reward (Friese et al., 2012; Derrick, 2013) could significantly improve participants’ self-control level in subsequent tasks. Therefore, we could conclude that delivering rewards on time is an effective way to improve people’s interest in some tasks as well as to motivate the persistence of engaging in activities.

Furthermore, the findings of the present study—that additional rewards (e.g., bonuses) increase rather than decrease intrinsic motivation—appear to contradict the studies about the “overjustification effect” (Lepper, 1981; Tang and Hall, 1995). However, there are actually two different reaction mechanisms. The overjustification effect refers to giving extra rewards to the participants in an activity, undermining intrinsic motivation by reducing the activity-goal association. The studies related to the “overjustification effect” compared the presence vs. absence of rewards. Instead, in this research, participants received an extra reward apart from a basic reward. The present study varied the delivery time of extra rewards, different from the studies about the “overjustification effect.” Therefore, in the context of the present study, basic rewards already existed; thus, the extra rewards could not dilute the intrinsic motivation or the good experience of the activity itself. In contrast, due to the immediacy of extra rewards, immediate extra rewards may help to stimulate and enhance the intrinsic motivation as well as the positive experience of the activity itself.

### Innovation

This study was innovative in some respects. First, the present study had a novel research perspective. Previous studies on the relationship between intrinsic motivation and external rewards have mainly focused on how the presence of external rewards influence intrinsic motivation intensity. Most studies have reached a relatively consistent conclusion: imposing external incentives will weaken participants’ intrinsic motivation. Additionally, researchers have developed many classical theories, such as the Deci effect (Deci, 1971), dilution effect (Ying et al., 2007; Orehek et al., 2012), and overjustification effect (Lepper, 1981). In this study, the factor we focused on was the change from “the presence of external rewards” to “the extra reward timing.” We focused on whether the timing of paying different extra rewards would impact people’s intrinsic motivation. The results showed that delivering immediate extra rewards can significantly enhance participants’ intrinsic motivation, indicating that the effect of external incentives weakening intrinsic motivation could be avoided. This study provided strong support for the position that immediate rewards enhance intrinsic motivation, which also has significant implications for in-depth studies of the triggering and working mechanisms of intrinsic motivation.

Another innovation of this study lies in creating new experimental schemes. The research of Woolley and Fishbach (2018) initially showed that providing an immediate bonus could increase participants’ intrinsic motivation. We believed their study did not rule out the interference of the misattribution effect of extrinsic motivation. Based on the different time-dependent characteristics of intrinsic and extrinsic motivation, we improved the experimental paradigms of Woolley’s study by (1) changing the time point of measuring intrinsic motivation and (2) temporarily changing the reward timing during the experiment. The present study ruled out the interference of the misattribution effect of extrinsic motivation and provided strong empirical evidence for the conclusion of prior research, that immediate external rewards enhance intrinsic motivation.

This research also has important value for practical fields, such as employee management and the realization of individual goals. For instance, for enterprise managers, compared with giving employees a massive bonus at the end of the year, smaller but more frequent bonuses after employees complete their work at different times throughout the year may perhaps have a better effect on stimulating employees’ interest and enthusiasm for work. Moreover, by providing more frequent and immediate rewards (such as practical and special gifts), marketers can constantly strengthen users’ intrinsic motivation to use their products as well as improve their loyalty to the product brand.

### Limitations and Prospects

In the present study, through four experiments, we eliminated the potential interference of the extrinsic motivation misattribution effect and obtained some interesting findings. However, there were still some limitations. (1) The applicability of the conclusions of the present research is of limited scope. In some cases, people may be more inclined to obtain delayed satisfaction than to obtain immediate rewards, such as enjoying a perfect holiday or tasting good wine (Loewenstein, 1987). In these cases, immediate rewards may not be helpful to increase intrinsic motivation. (2) We mainly used a self-report instrument to measure the dependent variable. Future research can use more methods, such as ERP, fMRI, and other neuroimaging methods, to add new evidence at the neural level.

## Conclusion

Based on excluding the interference of the misattribution effect of extrinsic motivation, the current study focused on whether immediate extra rewards could increase intrinsic motivation. By conducting three studies, the present study systematically demonstrated that an immediate bonus could enhance participants’ intrinsic motivation. Furthermore, the present research was of great theoretical and practical significance for future studies exploring the occurrence and working mechanisms of intrinsic motivation as well as developing nudging measures to stimulate or enhance intrinsic motivation.

## Data Availability Statement

The original contributions presented in the study are included in the article/Supplementary Material, further inquiries can be directed to the corresponding author.

## Ethics Statement

The studies involving human participants were reviewed and approved by Human Research Ethics Committee of South China Normal University. The patients/participants provided their written informed consent to participate in this study.

## Author Contributions

YL and YY developed the conception of the study, conducted the experiments, and drafted the manuscript. XB participated in the data analysis and some revision work. YC provided critical revisions in the final version of the manuscript. LM gave important advice throughout the whole study. All authors contributed to manuscript revision, read, and approved the submitted version.

## Conflict of Interest

The authors declare that the research was conducted in the absence of any commercial or financial relationships that could be construed as a potential conflict of interest.

## Publisher’s Note

All claims expressed in this article are solely those of the authors and do not necessarily represent those of their affiliated organizations, or those of the publisher, the editors and the reviewers. Any product that may be evaluated in this article, or claim that may be made by its manufacturer, is not guaranteed or endorsed by the publisher.
