# Supplementary material for: Do Immediate External Rewards Really Enhance Intrinsic Motivation?
Source: Front Psychol. 2022 May 16;13:853879. doi: 10.3389/fpsyg.2022.853879 (PMC9150741; doi:10.3389/fpsyg.2022.853879)
Supplement: Supplementary file 2 [file Table_2.DOCX]

# Appendix

Appendix A: Intrinsic Motivation Questionnaire

Hello!

There are seven questions in this questionnaire,.Please read each item carefully and choose the score(score:1-7, larger number represents larger intensity.) that best suits your real situation. We will keep your answer strictly confidential for you. There is no right and wrong answer, as long as it can reflect your real psychological activity when you conducted the reading task,If you have any questions or need help when answering, please feel free to ask the experimenter in time. Thank you for your cooperation!

⑴Do you like the content of the reading? 1 = Not satisfied at all，7 = Extremely satisfied

⑵Do you think this reading task is interesting? 1 = Not interesting at all，7 = Extremely interesting

⑶ Do you think this reading task is more like work or fun? 1 = More like work，7 = More like fun

⑷Do you think this reading task is boring? 1 = Not boring at all, 7 = Extremely boring

⑸I really get great pleasure in the reading. 1 = Extremely disagree，7 = Extremely agree

⑹I really enjoy in this reading. 1 = Extremely disagree，7 = Extremely agree

⑺Are you willing to continue reading without extra reward? 1= Extremely unwilling，7 = Extremely willing

Appendix B: Post-test Emotional Questionnaire

Hello!

There are three questions in this questionnaire. Please read each item carefully and choose the score(score:1-7, larger number represents larger intensity.) that best suits your real situation. We will keep your answer strictly confidential for you. There is no right and wrong answer, as long as it can reflect your real psychological activity when you conducted the reading task,If you have any questions or need help when answering, please feel free to ask the experimenter in time. Thank you for your cooperation!

Will changing the time of reward delivery affect your rating in subsequent task?

1. If there is any influence, please assume that there is no change in the time of award delivery, and re-grade the items of the scale.

⑴Do you like the content of the reading? 1 = Not satisfied at all，7 = Extremely satisfied

⑵Do you think this reading task is interesting? 1 = Not interesting at all，7 = Extremely interesting

⑶ Do you think this reading task is more like work or fun? 1 = work，7 = fun

⑷Do you think this reading task is boring? 1 = Not boring at all, 7 = Extremely boring

⑸I really get great pleasure in the reading. 1 = Extremely disagree，7 = Extremely agree

⑹I really enjoy in this reading. 1 = Extremely disagree，7 = Extremely agree

⑺Are you willing to continue reading without extra reward? 1= Extremely unwilling，7 = Extremely willing

2. If not, please briefly explain the reason why changing the reward delivery time influence your rating in subsequent task (what is the main basis of your scoring on the items? ).
